# *Mycobacterium tuberculosis* Induced Osteoblast Dysregulation Involved in Bone Destruction in Spinal Tuberculosis

**DOI:** 10.3389/fcimb.2022.780272

**Published:** 2022-04-06

**Authors:** Wenxin Ma, Weidong Jin, Xijing He, Yuhang Sun, Huquan Yin, Zili Wang, Shiyuan Shi

**Affiliations:** ^1^ Department of Spine Surgery, General Hospital of Ningxia Medical University, Ningxia, China; ^2^ Department of Spine Surgery, Xi’an International Medical Center Hospital Affiliated to Northwest University, Shaanxi, China; ^3^ Department of Orthopedics, Liaocheng Hospital of Traditional Chinese Medicine, Liaocheng, China; ^4^ Department of Biochemistry, Inteliex Biotechnology Corp, Tampa, FL, United States; ^5^ Department of Orthopedics, Hangzhou Chest Hospital affiliated to Zhejiang University Medical College, Zhejiang, China

**Keywords:** *Mycobacterium tuberculosis*, osteoblast, bone destruction, CD40, antisense oligonucleotide

## Abstract

Disturbance of bone homeostasis caused by *Mycobacterium tuberculosis* (*Mtb*) is a key clinical manifestation in spinal tuberculosis (TB). However, the complete mechanism of this process has not been established, and an effective treatment target does not exist. Increasing evidence shows that abnormal osteoclastogenesis triggered by an imbalance of the receptor activator of NF-κB ligand (RANKL)/osteoprotegerin (OPG) axis may play a key role in the disturbance of bone homeostasis. Previous studies reported that RANKL is strongly activated in patients with spinal TB; however, the OPG levels in these patients were not investigated in previous studies. In this study, we investigated the OPG levels in patients with spinal TB and the dysregulation of osteoblasts caused by *Mtb* infection. Inhibition of the *Mce4a* gene of *Mtb* by an antisense locked nucleic acid (LNA) gapmer (Mce4a-ASO) was also investigated. Analysis of the serum OPG levels in clinical samples showed that the OPG levels were significantly decreased in patients with spinal TB compared to those in the group of non-TB patients. The internalization of *Mtb* in osteoblasts, the known major source of OPG, was investigated using the green fluorescent protein (GFP)-labeled *Mycobacterium* strain H37Ra (H37RaGFP). The cell-associated fluorescence measurements showed that *Mtb* can efficiently enter osteoblast cells. In addition, *Mtb* infection caused a dose-dependent increase of the CD40 mRNA expression and cytokine (interleukin 6, IL-6) secretion in osteoblast cells. Ligation of CD40 by soluble CD154 reversed the increased secretion of IL-6. This means that the induced CD40 is functional. Considering that the interaction between CD154-expressing T lymphocytes and bone-forming osteoblast cells plays a pivotal role in bone homeostasis, the CD40 molecule might be a strong candidate for mediating the target for treatment of bone destruction in spinal TB. Additionally, we also found that Mce4a-ASO could dose-dependently inhibit the *Mce4a* gene of *Mtb* and reverse the decreased secretion of IL-6 and the impaired secretion of OPG caused by *Mtb* infection of osteoblast cells. Taken together, the current finding provides breakthrough ideas for the development of therapeutic agents for spinal TB.

## Introduction

Tuberculosis (TB), a disease caused by *Mycobacterium tuberculosis* (*Mtb*), is one of the oldest infectious diseases in history. To date, TB is still one of the top 10 leading causes of death worldwide. Moreover, bone and joint tuberculosis (BJTB) accounts for about 3%–7% of all tuberculosis cases, among which over 70% are spinal tuberculosis (Khanna and Sabharwal, 2019). Spinal tuberculosis is a condition characterized by an enormous resorption of the spinal vertebrae due to *Mtb* infection. It is described as a persistent inflammatory destruction of the spinal bones, essentially incited by mycobacterial disease of the spinal cavity, and is believed to be initiated by the odd activation of osteoclasts in the bone tissue, which leads to inflammatory bone destruction, crack, and breakdown of the vertebrae, bringing about gigantic kyphotic distortions (Haynes, 2004; Hoshino et al., 2014).

Bones are composed of a variety of cell types, namely, osteoclasts, osteoblasts, osteocytes, and bone lining cells (Tobeiha et al., 2020). Osteoclasts originate from hematopoietic stem cells (HSCs, the macrophage lineage of hematopoietic stem cells), while osteoblasts originate from mesenchymal stem cells (MSCs) through certain stages, for example, osteoprogenitors and pre-osteoblasts (Datta et al., 2008; Thompson et al., 2012). In addition, bone homeostasis is maintained by the harmony between bone resorption by osteoclasts and bone deposition by osteoblasts (Eriksen, 2010; Rowe et al., 2021). Osteoclasts comprise a class of multinucleated bone destruction cells formed by the cytoplasmic combination of their mononuclear forerunners, which are in the myeloid heredity of hematopoietic cells that additionally bring about macrophages (Boyce and Xing, 2007). Activated osteoclasts launch proteolytic enzymes, which smash connective tissues in bones. They additionally secrete some acids that unravel the mineral phase of bones (Mundy, 2007). The formation and activation of osteoclasts require the expression of the osteoprotegerin ligand (OPGL) [or receptor activator of NF-κB ligand (RANKL)] in osteoblasts, which can be induced by calciotropic factors such as vitamin D3, interleukin 1 (IL-1), and tumor necrosis factor alpha (TNF-α). Once OPGL is induced, it then binds to RANKL on osteoblast precursors, leading to the maturation and activation of osteoblasts (Burgess et al., 1999; Hsu et al., 1999; Kong et al., 2000). Osteoprotegerin (OPG), which is secreted from osteoblasts, features as a soluble decoy receptor to OPGL and competes with the receptor activator of NF-κB (RANK) for binding to the OPGL. Consequently, OPG functions as an inhibitor of osteoclast maturation and osteoclast activation. Previous genetic and purposeful experiments pointed out that the stability between OPGL–RANK signaling and the degree of biologically energetic OPG alter the improvement and activation of osteoclasts and bone metabolism (Bucay et al., 1998; Li et al., 2000). So far, research on this topic has continuously indicated that the disturbance of bone homeostasis is caused by the over-activation of osteoblasts through the regulation of OPGL–RANK (Simonet et al., 1997; Morony et al., 1999; Tanaka et al., 2005; Baud’huin et al., 2007). Moreover, *Mtb* infection of osteoblasts will increase the secretion of chemokines (such as IL-8, IP-10, and MCP-1), which will subsequently affect the recruitment of leukocytes and ultimately contribute to bone destruction. Therefore, chemokines derived from *Mtb*-infected osteoblasts may play an important role in the development of tuberculous and staphylococcal osteomyelitis (Wright and Friedland, 2002). These findings reinforced the general belief that osteoblasts play a pivotal role in the maturation and activation of osteoclasts for bone resorption locales. Likewise, osteoblasts could control bone resorption by the discharge of OPG and RANKL, and the RANK/OPG ratio may be the determinant factor for bone homeostasis. Pieces of evidence from other studies have suggested that the RANK–RANKL pathway is strongly activated and that the tissue expression of OPG is not fully induced in spinal TB bone specimens (Izawa, 2015). Treatment of recombinant *M. tuberculosis* (r-Mt) 10-kDa co-chaperonin (cpn10) triggered a significantly downregulated level of OPG and upregulated level of RANKL in human osteoblast cells (Zhang et al., 2015). It should also be noted that *Mtb* could inhibit the complement receptor (CR)-mediated Ca^2+^ signaling (Malik et al., 2000) and could lead to the impairment of OPG expression and secretion (Bergh et al., 2004). Infection of mice by acute administration of microbial results in elevated serum levels of OPG, which causes disruption of beta cell homeostasis by inhibiting glucose-stimulated insulin secretion (GSIS) (Kuroda et al., 2016). These findings suggest that there is great interest in investigating the circulating OPG levels in spinal TB patients.

One of the hallmarks of spinal TB is granuloma formation. Granuloma formation is a specific provocative reaction caused by specific microbes such as *Mycobacterium* (Shibuya et al., 2005). It is usually regarded as a host protective mechanism toward irritants or persistent contamination and occurs as an outcome of the failure of the host to defend against invading pathogens. Various cell types are involved in granuloma formation, such as macrophages, T cells, and epithelioid cells; however, the presence of multinucleated Langhans giant cells (LGCs) is the main feature of granulomatous inflammation (Sakai et al., 2012). Nevertheless, the complete mechanism of LGC formation is still not fully elucidated. The generally accepted theory is that cytokines (e.g., IFN-γ, IL-3, and IL-4) secreted from T cells may play pivotal roles in this process (Enelow et al., 1992; Takashima et al., 1993). Additionally, an antibody inhibition experiment showed that CD40–CD40 ligand (CD40L, or CD154) interaction and interferon gamma (IFN-γ) are required for LGC formation and that soluble CD40L (sCD40L) and IFN-γ could completely replace the role of T cells (Sakai et al., 2012). These facts suggest that the CD40–CD40L axis has a critical role in the formation of LGCs. The interactions between CD40 and CD154, especially the ability of CD154-expressing T lymphocytes to ligate CD40-expressing cells, have been embroiled as being basic for the commencement of T-lymphocyte–subordinate humoral and immune reactions (Stout et al., 1996; Calderhead et al., 2000; Netea et al., 2002). These interactions provide strong activation signals for antigen-presenting cells, allowing T lymphocytes to strongly affect the activity of CD40-expressing cells; the absence of these interactions significantly impairs the cell-mediated immune response (Calderhead et al., 2000; van Kooten, 2000). The traditionally recognized feature of osteoblasts is to synthesize type I collagen in order to catalyze the calcification of the bone matrix. However, pieces of evidence from recent studies have suggested that, following interplay with external bacteria, bone-forming osteoblasts have been proven to possess a surprising potential to upregulate the expressions of cytokines and chemokines (Taichman and Emerson, 1994; Bost et al., 1999; Graves et al., 1999; Bost et al., 2001), which tend to increase the T-lymphocyte-mediated inflammatory responses. In the current study, we investigated the expression of functional CD40 in human osteoblasts following interplay with *Mtb* and the changes in the secretion of inflammatory cytokines, which may influence the T-lymphocyte-mediated inflammatory responses in spinal TB.

*M. tuberculosis* has four homologues of mammalian cell entry (mce) operons. Among them, mutation in the mce4 operon resulted in growth defects and in the reduced survival of *Mycobacterium*-infected mice (Saini et al., 2008). Moreover, mce genes have been proven to play essential roles in *Mtb* pathogenicity by modulating the host immune response (Checkley et al., 2011). In this study, we designed a locked nucleic acid (LNA)–DNA–LNA gapmer antisense specific to *Mce4a* in *Mtb* to examine whether it could inhibit *Mycobacterium*.

## Materials and Methods

### Clinical Samples

This study included clinical samples from seven adult patients with spinal TB (four men and three women; average age, 61.3 years) and eight normal volunteer donors (four men and four women; average age, 59.2 years). The baseline characteristics of the study subjects are shown in **Table 1**. The ethics of this study was reviewed by the Research Ethics Committee of the General Hospital of Ningxia Medical University, which considered it in compliance with the principles of research ethics and approved it (2014). All study participants gave informed consent.

**Table 1:** Baseline characteristics of the study participants.

	Non-spinal TB controls	Spinal TB patients
Age (years)	59.2 ± 8.8	61.3 ± 14.2
Sex: male/female (%)	50/50	50/50
Laboratory test	Negative	Xpert positive, TB DNA/RNA positive
ESR	N/A	53.3 ± 24.8
Surgery	None	None
Diagnosis	Asymptomatic	Low back pain, lumbar tuberculosis

TB, tuberculosis; ESR, erythrocyte sedimentation rate; N/A, not applicable.

### Mammalian Cell Culture

Human bone tissue-derived osteoblasts (CRL-11372) [American Type Culture Collection (ATCC), Manassas, VA, USA] were maintained in the logarithmic phase of growth in a 1:1 mixture of Ham’s F12 medium/Dulbecco’s modified Eagle’s medium (Gibco-BRL, Grand Island, NY, USA), with 2.5 mM l-glutamine (without phenol red), 0.3 mg/ml G418, and supplemented with heat-inactivated 10% fetal bovine serum (Gibco-BRL) at 34°C in a 5% CO_2_–95% air-humidified incubator. Human Jurkat T-lymphocyte leukemia cell lines (TIB-152; ATCC) were maintained in the logarithmic phase of growth in RPMI-1640 medium (Gibco-BRL) supplemented with heat-inactivated 10% fetal bovine serum (Gibco-BRL) at 37°C in a 5% CO_2_–95% air-humidified incubator.

### Establishment of GFP-Expressing *M. tuberculosis* (H37Ra-GFP)

The *Mycobacterium* Strong Promoter (MSP) and green fluorescent protein (GFP) containing the pGFPHYG2 plasmid was a gift from Lalita Ramakrishnan (Addgene plasmid #30173, RRID: Addgene_30173; http://n2t.net/addgene:30173). *Mtb* strain H37Ra (25177; ATCC) was maintained in the medium log phage in Middlebrook 7H9 broth with ADC enrichment at 37°C. pGFPHYG2 was introduced directly into the *Mtb* strain by electroporation using a Gene Pulser electroporator (Bio-Rad, Richmond, CA, USA). Electroporation of plasmid DNA was performed at settings of 2.5 kV, 25 µF, 1,000 Ω (Roberts et al., 2004).

### Measurement of Osteoprotegerin Levels

The levels of OPG were detected from human serum and cell culture medium and were measured with enzyme-linked immunosorbent assay (ELISA) using a commercially available kit (Boster Bio, Wuhan, China) according to the manufacturer’s instruction. The OPG produced by osteoblast cells was normalized to the cell densities with the CellTiter-Blue^®^ assay (Promega, Madison, WI, USA).

### Reverse Transcription Polymerase Chain Reaction

For mammalian cells, total RNA was prepared using the TRIzol reagent according to the manufacturer’s protocol (Invitrogen, Carlsbad, CA, USA), and single-strand complementary DNA (cDNA) was synthesized from the RNA in a reaction mixture containing optimum blend of oligo(dT) primers and iScript reverse transcriptase (Bio-Rad, Richmond, CA, USA). Quantitative reverse transcription PCR (qRT-PCR) amplifications were performed using the rEVAltion 2× qPCR Master Mix (Empirical Bioscience, Grand Rapids, MI, USA) in an MyIQ2 Real-Time PCR Detection System (Bio-Rad) following the manufacturer’s protocol. To determine the specificity of amplification, melting curve analysis was applied to all final PCR products. The relative amount of target messenger RNA (mRNA) was calculated using the comparative threshold cycle method by normalizing the target mRNA threshold cycle to that for glyceraldehyde-3-phosphate dehydrogenase (GAPDH). For bacterial cells, total RNA was prepared using FastPrep^®^ instruments from MP Biomedicals (Irvine, CA, USA) according to the manufacturer’s protocol. The gene-specific primers used in this study are listed in **Table 2**.

**Table 2:** Gene-specific primers used in PCR amplification.

Gene	Forward primer (5′–3′)	Reverse primer (5′–3′)
*CD40*	AAGAAGGCTGGCACTGTA	GATGACACATTGGAGAAGAAG
*Mce1a*	CTCGTAGTTGCCTTGGTAT	CGGAATCAGGTGGATGTAA
*Mce2a*	GTTGGTGCTCGTATTGGT	CGCTGCTGTTGAGGAATT
*Mce3a*	GGCAAGGTGAACGGATAG	CATAGGTGTCGGTGAAGTT
*Mce4a*	GAGTAGTCGGAGGTCACT	CATCGGTCTGTCTAATAACG
*GAPDH*	TATGACAACAGCCTCAAGAT	AGTCCTTCCACGATACCA
16S RNA	TCCCGGGCCTTGTACACA	CCACTGGCTTCGGGTGTTA

### Measurement of Secreted Interleukin 6

The collected cell culture medium was used to measure the levels of released IL-6. The IL-6 levels in the cell culture medium were measured with ELISA using a commercially available kit (BioVision, Milpitas, CA, USA) according to the manufacturer’s instruction. The results were normalized to the cell densities using the CellTiter-Blue^®^ assay (Promega).

### Quantification of *M. tuberculosis* Internalization Into Host Cells

Mycobacterium internalization was measured as previously described (Ashiru et al., 2010), with some modifications. Briefly, GFP-labeled *Mtb* was added to each cell culture at multiplicities of infection (MOIs) of 1:1,000, 1:100, and 1:10, and extracellular bacteria were removed by amikacin (200 μg/ml) treatment for a total of 1 h. The internalization of *Mtb* was evaluated by measuring the cell-based fluorescence using a SpectraMax Gemini EM fluorescence microplate reader. For imaging of internalized cells, fluorescence-labeled *Mtb* was cultured in Middlebrook 7H9 with OADC. Osteoblast cells were infected with the culture medium or *Mtb* for 5 h at an MOI of 20, and non-infected bacteria were removed by washing with phosphate-buffered saline (PBS) three times. The image was captured using a fluorescence microscope at an original magnification of ×200.

### Locked Nucleic Acid Contained Antisense Oligonucleotides

The *Mce4a*-specific antisense oligonucleotide containing LNA (LNA–DNA–LNA gapmer), which can effectively induce RNase H activation, had the following sequence: 5′-(TYE563)+G+G+A+A+CGGGACGACT+T+C+T+G+A-3′ (“+” denotes the site of LNA). The 5′-end of the antisense gapmer was labeled with a TYE563 fluorescence dye.

### Quantification of Colony Forming Units

*Mtb* was added to the cell culture in the presence or absence of Mce4a-ASO at various MOIs for indicated times, and extracellular bacteria were removed by amikacin (200 μg/ml) treatment. The cells were lysed using a 0.1% TritonX-100-based lysis buffer and plated in a 7H11 agar plate. The colonies were counted using ImageLab^®^ software (Bio-Rad, Hercules, CA, USA).

### Statistical Analysis

Statistical significance was evaluated using GraphPad Prism 6.0 statistics software program (GraphPad Software, Inc., San Diego, CA, USA). Between-group comparisons were analyzed using two-tailed unpaired Student’s *t*-test or two-way analysis of variance (ANOVA). Statistical significance was set at *p* < 0.05.

## Results

### Circulating Osteoprotegerin Levels Were Decreased in Spinal TB Patients

**Figure 1:**
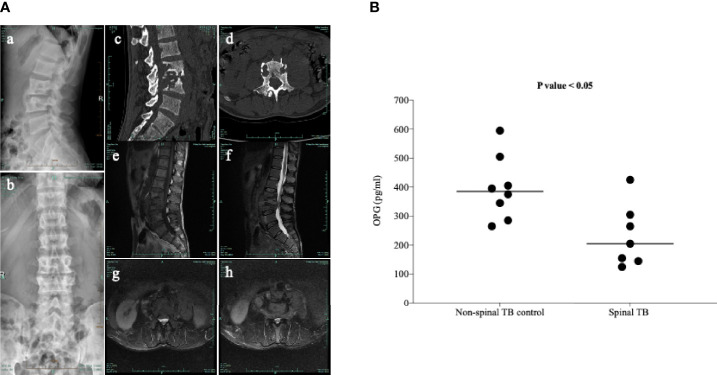
Typical clinical manifestations of spinal TB in the study participants. **(A)** Typical radiology of a patient with spinal TB used in this study. (*a*, *b*) X-ray front and side positions suggesting the destruction of lumbar 3 and 4 vertebral bodies. (*c*, *d*) CT sagittal view showing bone destruction in the lumbar 2, 3, and 4 vertebrae. The CT axis showed severe damage to the vertebral body. (*e*–*h*) T1 and T2 images and axial position of MRI indicating bone destruction of the lumbar 2, 3, and 4 vertebrae, accompanied by paravertebral abscess formation. **(B)** Measurement of the circulating OPG levels in patients with spinal TB using a commercially available ELISA kit. Means without a common letter differ. *p* < 0.05.

It is well known that osteoclast differentiation could be inhibited by OPG binding to the RANKL and that changes in the OPG/RANKL ratio determine osteoclastogenesis (Ominsky et al., 2008). Previous studies showed that the expression of RANKL is strongly activated in patients with spinal TB (Izawa, 2015); however, there is no report on whether the levels of OPG changed in these patients. The clinical characteristics of patients are summarized in **Table 1**, and typical specimen magnetic resonance imaging (MRI) scans are shown in **Figure 1A**. We first investigated the circulating OPG levels in patients with spinal TB. As shown in **Figure 1B**, the circulating OPG levels were significantly decreased in patients with spinal TB compared to those in non-spinal TB controls.

### *M. tuberculosis* (H37Ra-GFP) Internalized and Changed the OPG/RANKL mRNA Ratio in Osteoblasts

**Figure 2:**
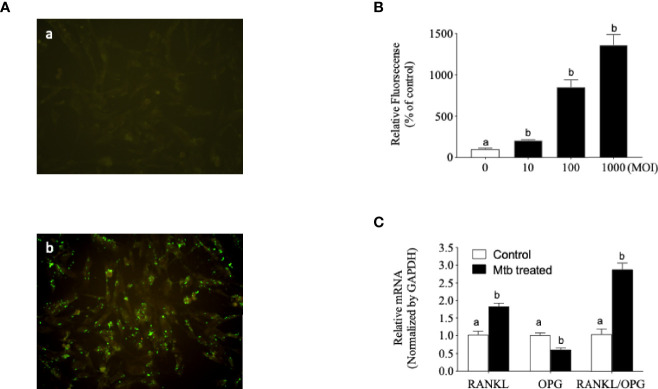
*Mycobacterium tuberculosis* (*Mtb*) internalized human osteoblast cells and changed the receptor activator of NF-κB ligand (RANKL)/osteoprotegerin (OPG) ratio. **(A)** Fluorescence-labeled *Mtb* were cultured in Middlebrook 7H9 with OADC. Osteoblast cells were infected with the culture medium or *Mtb* for 5 h at a multiplicity of infection (MOI) of 20, and non-infected bacteria were removed by washing with phosphate-buffered saline (PBS) three times. (*a*) Control. (*b*) *Mtb* infection. Images are shown at ×200 original magnification. **(B)** Fluorescence-labeled *Mtb* were co-cultured in accordance with the experimental procedure described in *Materials and Methods*. The internalized *Mtb* was measured based on the increased fluorescence density. **(C)** Single-stranded cDNA was synthesized from the total RNA from *Mtb*-infected osteoblast cells, and RT-PCR was performed with the gene-specific primers described in *Materials and Methods*. Means without a common letter differ. *p* < 0.05.

OPG, also known as osteoclastogenesis inhibitory factor (OCIF), is a glycoprotein that is expressed by various cells in humans, including osteoblasts, epithelial cells, endothelial cells, B cells, and CD4^+^ lymphocytes (Sordillo and Pearse, 2003; Chakravarti et al., 2008). Based on the evidence that OPG may be involved in spinal TB, we investigated the internalization of *Mtb* and the change in the OPG/RANKL ratio in osteoblast cells. For quantification of *Mtb* internalization into host cells, GFP-expressing *Mtb* strains were introduced into human osteoblast cells. Cell-based fluorescence analysis indicated that *Mtb* was effectively internalized into human osteoblast cells (**Figures 2A, B**). Treatment of *Mtb* also changed the OPG/RANKL mRNA ratio (**Figure 2C**).

### Functional CD40 Was Induced by *Mtb* Treatment in Osteoblasts

**Figure 3:**
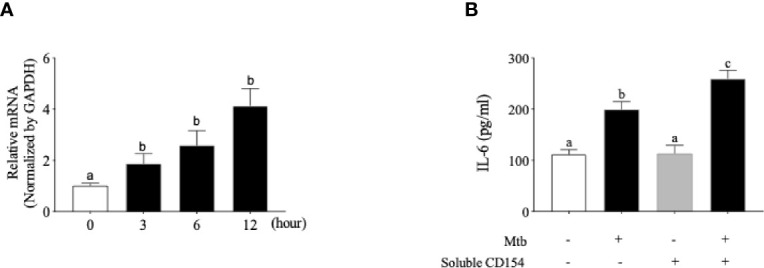
Functional CD40 molecule is induced by *Mycobacterium tuberculosis* (*Mtb*) infection in human osteoblast cells. **(A)** Quantitative real-time PCR analysis for CD40 performed on single-stranded cDNA synthesized from the total RNA from *Mtb*-infected osteoblast cells. **(B)** Interleukin 6 (IL-6) secretion after ligation of CD40 by soluble CD154 in osteoblast cells. After *Mtb* infection, osteoblast cells were treated with soluble CD154, and the cell culture supernatant was collected 24 h later for the analysis of IL-6 secretion. The level of IL-6 was measured using a commercially available ELISA kit. Means without a common letter differ. *p* < 0.05. Groups that do not share a lower case letter are significantly different.

T-lymphocyte and macrophage infiltration is often observed in the disturbance of bone homeostasis (Taubman and Kawai, 2001). Our next question was focused on whether the *Mtb* internalized in osteoblasts acts as a co-stimulatoryfactor of T lymphocytes in terms of CD40 induction in order to influence the bone homeostasis disturbance caused by *Mtb*. To examine whether *Mtb* infection induces the CD40 mRNA, osteoblast cells were treated with medium log-phase-maintained *Mtb* at an MOI of 1:100 for a total of 12 h. As shown in **Figure 3A**, the CD40 mRNA was increased in a time-dependent manner. We subsequently examined whether this increased expression of CD40 is functional. The functionality of CD40 was assessed based on the secretion changes of the cytokine IL-6. After infection with *Mtb*, osteoblast cells were treated with soluble CD154/TRAP fusion protein for the ligation of CD40. As shown in **Figure 3B**, the ligation of CD40 caused a significantly increased secretion of IL-6 by *Mtb* infection in osteoblasts.

### *Mce4a*-Specific Antisense LNA Gapmers (Mce4a-ASO) Reversed the Cytokine Release and Decreased the OPG Production in *Mtb*-Infected Osteoblasts

**Figure 4:**
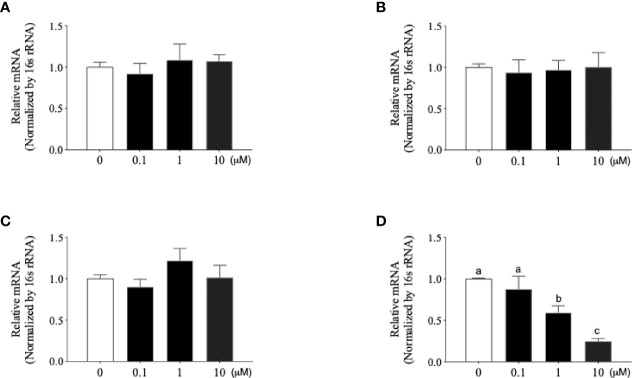
Newly designed *Mce4a* antisense locked nucleic acid (LNA) gapmer (Mce4a-ASO) specifically downregulates the expression of *Mce4a* in *Mycobacterium tuberculosis* (*Mtb*). *Mtb* was incubated with various concentrations (0.1–10 µM) of Mce4a-ASO for 48 h. Gene expression was measured by real-time PCR using gene-specific primers. **(A–D)** Expression levels of *Mce1a* **(A)**, *Mce2a* **(B)**, *Mce3a* **(C)**, and *Mce4a* **(D)**. Means without a common letter differ. *p* < 0.05. Groups that do not share a lower case letter are significantly different.

**Figure 5:**
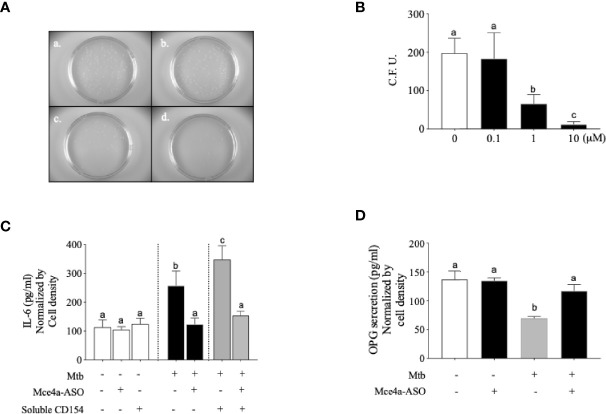
Mce4a-ASO reduced *Mycobacterium tuberculosis* (*Mtb*) survival and cytokine secretion in human osteoblast cells. Osteoblast cells were incubated with *Mtb* in the presence or absence of Mce4a-ASO (0–10 µM) for 48 h. **(A)** Representative images of the colony forming unit (CFU) Petri dishes. (*a*) *Mtb* only. (*b*) *Mtb* infection with 0.1 µM Mce4a-ASO. (*c*) *Mtb* infection with 1 µM Mce4a-ASO. (*d*) *Mtb* infection with 10 µM Mce4a-ASO. **(B)** The number of bacterial colonies was determined using ImageLab^®^ software. **(C)** The secretion level of interleukin 6 (IL-6) was measured using a commercially available ELISA kit. **(D)** Osteoprotegerin produced by osteoblasts was measured using a commercially available ELISA kit according to the manufacturer’s protocol. Means without a common letter differ. *p* < 0.05.

Based on the various responses of *Mtb*-infected osteoblasts, we wondered whether inhibition of *Mycobacterium* activity in osteoblasts could affect these responses. Firstly, we designed the *Mce4a*-specific antisense LNA gapmers (Mce4a-ASO). To confirm the specificity of Mce4a-ASO, we first examined the effects of Mce4a-ASO on the mRNA expressions of the mce family genes in *Mtb* culture. When the mRNA levels of the *Mce1a*, *Mce2a*, *Mce3a*, and *Mce4a* genes were measured with real-time PCR using gene-specific primers, we found that the mRNA expression of *Mce4a* decreased significantly in a dose-dependent manner, but those of *Mce1a*, *Mce2a*, and *Mce3a* were not significantly changed by Mce4a-ASO treatment (**Figure 4**). The *Mce4a* gene is related to the invasion of mycobacteria into host cells and is important to the survival of bacilli in cells (Saini et al., 2008). Therefore, we subsequently investigated the effects of Mce4a-ASO on osteoblast response to *Mtb* internalization. After incubation of the *Mtb*-infected osteoblast cells with or without Mce4a-ASO (0–10 µM) for 48 h, we measured the number of intracellular bacteria by quantification of the colony forming units (CFU). CFU analysis indicated that Mce4a-ASO markedly decreased the intracellular numbers of mycobacteria (**Figures 5A, B**). In addition, the measurement of cytokines in the cell culture supernatant showed that the cytokine release (IL-6) caused by *Mtb* infection was significantly decreased by Mec4a-ASO co-treatment in osteoblasts (**Figure 5C**). The enhanced IL-6 release by co-treatment with CD154 was also reversed by Mec4a-ASO co-treatment in osteoblast cells (**Figure 5C**). To determine whether the decreased OPG production in *Mtb*-infected osteoblasts was affected by Mec4a-ASO treatment, the production of OPG was measured using ELISA. As shown in **Figure 5D**, the decreased production of OPG due to *Mtb* infection was reversed by Mec4a-ASO co-treatment in osteoblast cells (**Figure 5D**).

## Discussion

In this study, we investigated the involvement of osteoblasts in *Mtb*-triggered disturbance of bone homeostasis in spinal TB. Analysis of the circulating OPG content in patients with spinal TB showed significantly decreased levels when compared to those in non-spinal TB patients. In *in vitro* studies using human osteoblast cells, *Mtb* exhibited efficient internalization into cells and triggered functional CD40 expression. Treatment with *Mce4a*-specific antisense LNA oligonucleotides significantly decreased the bacterial load in *Mtb*-infected osteoblasts, and the key cytokine (IL-6) release was also significantly decreased by Mce4a-ASO co-treatment.

With thousands of years of infection history, tuberculosis remains a modern and life-threatening health problem. According to the WHO report, an estimated 10 million incidents of TB cases and 1.5 million TB deaths occurred globally in 2018. Bone and joint involvement accounts for approximately 10% of extrapulmonary tuberculosis cases, and the spine is the most commonly affected site. Progressive bone destruction will ultimately lead to vertebral collapse and kyphosis in patients with spinal TB. Several lines of evidence have suggested that the RANLK/RANK/OPG system contributes to bone homeostasis disturbance (Baud’huin et al., 2007; Boyce and Xing, 2007; Ominsky et al., 2008; Tobeiha et al., 2020). In the present study, the circulating OPG levels were significantly decreased in patients with spinal TB, and it has been known that RANKL is strongly activated in these patients (Izawa, 2015). Additionally, within normal physiological conditions, the osteoblast-derived RANKL interacted with the RANK of the osteoclast precursors and through TRAF6/NF-κB/NFTATC1 to activate the OPG gene. Moreover, the activated OPG featured as a soluble decoy receptor to the OPGL and competed with RANK for binding to the OPGL. Therefore, OPG functioned as an inhibitor of osteoclast maturation and osteoclast activation. Our current data showed that the internalized *Mtb* could disrupt RANKL/OPG homeostasis in human osteoblast cells. Therefore, these results suggest that the decreased ratio of OPG/RANKL in osteoblast cells might have contributed to the bone homeostasis disturbance in patients with spinal TB.

Osteoblasts are specialized mesenchymal bone marrow stromal cells that synthesize type 1 collagen, coordinate the mineralization of the skeleton, and regulate osteoclast activation (Dirckx et al., 2019). When bone-forming osteoblasts interact with bacteria, the release of cytokines and chemokines are increased. An increased cytokine release could enhance the response of T lymphocytes to inflammation (Schrum et al., 2003). The interaction of CD40 and CD154 (the CD40 ligand) is considered as an essential mechanism of the T-lymphocyte-mediated immune response to pathogens (Noelle, 1996). Engaging CD40 on dendritic cells using the CD40 agonist significantly enhanced the antigen-specific Th_17_ response in mice, which means that the CD40–CD154 pathway plays a key role in adaptive immune response against tuberculosis (Sia et al., 2017). In addition to the inflammation-related immune response, the CD40–CD154 axis is necessary for LGC formation (Sakai et al., 2012), which is an important signature manifestation of spinal TB. These previous findings suggest that CD154-expressed T-lymphocyte-mediated interaction with CD40-expressed osteoblasts is a possible cause of the bone homeostasis disturbance brought about by *Mtb* infection. In the current study, we demonstrated that the expression of CD40 was significantly enhanced by the internalized mycobacterium, and the functionality of the enhanced CD40 was exhibited by co-treatment with soluble CD154 fusion protein. IL-6 secretion from osteoblasts was significantly augmented by soluble CD154, which means that the increased CD40 is functional in *Mtb*-infected osteoblasts and highly suggests that the enhanced functional CD40 is a possible mechanism of osteoblast-induced cytokine release.

The LNA-based antisense oligonucleotide is a new-generation methodology due to its characteristic of nuclease resistance, stability in plasma (Veedu and Wengel, 2009), and its ability to be delivered to cells without chemical transfection (Zhang et al., 2011). Previous studies showed that the mce genes have pivotal roles in the pathogenicity of *Mtb* by modulating the immune response of the host (Schrum et al., 2003; Checkley et al., 2011), and the reduced expression of the *Mce4a* gene of *Mtb* resulted in a significantly reduced intracellular survival of bacteria. In this study, we designed an *Mce4a*-specific LNA–DNA–LNA gapmer antisense (Mce4a-ASO) to modulate the effects of *Mtb* on host cells. Treatment with Mce4a-ASO caused a significantly reduced survival of *Mtb*, and its cytokine release also significantly changed inside osteoblast cells. These results suggest that inhibiting the *Mce4a* gene using an LNA-based gapmer is an effective strategy to modulating the behavior of *Mtb* in the disease progression of spinal TB.

Although the mechanism by which *Mtb* induces bone homeostasis disturbance is complex, a variety of molecules have been proposed as targets for therapeutic intervention. In the present study, we provided not only exploratory insights into understanding the role of *Mtb*-infected osteoblasts in bone homeostasis disturbance in spinal TB but also breakthrough ideas for the effective treatment of human spinal TB. Two potential limitations of this study are the relatively small total number of patients and the lack of evidence from *in vivo* model studies. However, this study provided substantial evidence of *Mtb*-infected osteoblasts in impaired bone homeostasis in spinal TB.

In subsequent studies, we will focus on constructing pRNA-3WJ nanoparticles that simultaneously contain CD40 RNA aptamers for targeting and *Mce4a*-specific ASO for modulating the behavior of *Mtb* and will investigate the effects on bone homeostasis disturbance in an animal model of spinal TB.

## Data Availability Statement

The original contributions presented in the study are included in the article/supplementary material. Further inquiries can be directed to the corresponding authors.

## Ethics Statement

The studies involving human participants were reviewed and approved by the Research Ethics Committee of the General Hospital of Ningxia Medical University. The patients/participants provided written informed consent to participate in this study.

## Author Contributions

WM: Conceptualization, Methodology. WJ: Investigation Methodology. XH: Investigation Methodology and Data curation. YS: Investigation Methodology. HY: Data curation, Writing- Original draft preparation. ZW: Funding acquisition, Supervision, Project administration, Writing- Reviewing and Editing. SS: Supervision, Project administration, Writing- Reviewing and Editing. All authors contributed to the article and approved the submitted version.

## Funding

This work was supported by a National Natural Science Foundation of China (Grant No. 81460336; Grant No. 81660370) and Ningxia youth talent promotion project.

## Conflict of Interest

Author HY is employed by Inteliex Biomedical Corporation.

The remaining authors declare that the research was conducted in the absence of any commercial or financial relationships that could be construed as a potential conflict of interest.

## Publisher’s Note

All claims expressed in this article are solely those of the authors and do not necessarily represent those of their affiliated organizations, or those of the publisher, the editors and the reviewers. Any product that may be evaluated in this article, or claim that may be made by its manufacturer, is not guaranteed or endorsed by the publisher.
